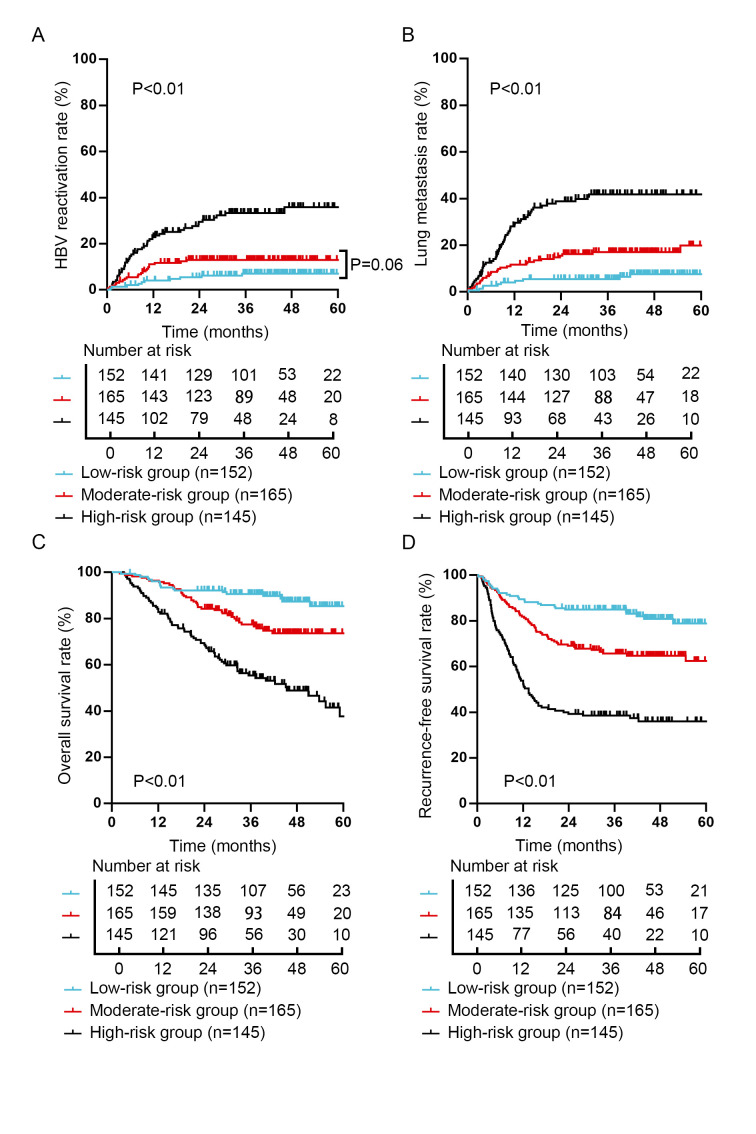# Post-transplant hepatitis B virus reactivation impacts the prognosis of patients with hepatitis B-related hepatocellular carcinoma: a dual-centre retrospective cohort study in China

**DOI:** 10.1097/JS9.0000000000001141

**Published:** 2024-02-09

**Authors:** Huigang Li, Di Lu, Jinyan Chen, Junchi Zhang, Jianyong Zhuo, Zuyuan Lin, Chenghao Cao, Wei Shen, Chiyu He, Hao Chen, Zhihang Hu, Yiyang Sun, Xuyong Wei, Li Zhuang, Shusen Zheng, Xiao Xu

**Affiliations:** a Zhejiang University School of Medicine; b Key Laboratory of Integrated Oncology and Intelligent Medicine of Zhejiang Province, Hangzhou; c The Fourth School of Clinical Medicine, Zhejiang Chinese Medical University, Hangzhou; d Department of Hepatobiliary and Pancreatic Surgery, Shulan (Hangzhou) Hospital, Hangzhou; e Department of Hepatobiliary and Pancreatic Surgery, The First Affiliated Hospital, Zhejiang University School of Medicine, Hangzhou; f National Center for Healthcare Quality Management in Liver Transplant, Hangzhou China

**Keywords:** hepatitis B virus reactivation, hepatocellular carcinoma recurrence, liver transplantation, lung metastasis

## Abstract

**Background:**

Highly active hepatitis B virus (HBV) is known to be associated with poor outcomes in patients with hepatocellular carcinoma (HCC). This study aims to investigate the relationship between HBV status and HCC recurrence after liver transplantation.

**Methods:**

The study retrospectively analyzed HCC patients undergoing liver transplantation in two centres between January 2015 and December 2020. The authors reviewed post-transplant HBV status and its association with outcomes.

**Results:**

The prognosis of recipients with hepatitis B surface antigen (HBsAg) reappearance (*n*=58) was poorer than those with HBsAg persistent negative (*n*=351) and positive (*n*=53). In HBsAg persistent positive group, recipients with HBV DNA reappearance or greater than 10-fold increase above baseline had worse outcomes than those without (*P*<0.01). HBV reactivation was defined as (a) HBsAg reappearance or (b) HBV DNA reappearance or greater than 10-fold increase above baseline. After propensity score matching, the 5-year overall survival rate and recurrence-free survival rate after liver transplantation in recipients with HBV reactivation were significantly lower than those without (32.0% vs. 62.3%; *P*<0.01, and 16.4% vs. 63.1%; *P*<0.01, respectively). Moreover, HBV reactivation was significantly related to post-transplant HCC recurrence, especially lung metastasis. Cox regression analysis revealed that beyond Milan criteria, microvascular invasion and HBsAg-positive graft were independent risk factors for post-transplant HBV reactivation, and a novel nomogram was established accordingly with a good predictive efficacy (area under the time‐dependent receiver operating characteristic curve=0.78, C-index =0.73).

**Conclusions:**

Recipients with HBV reactivation had worse outcomes and higher tumour recurrence rates than those without. The nomogram could be used to evaluate the risk of post-transplant HBV reactivation effectively.

## Introduction

Highlights

- The status of hepatitis B virus (HBV) activity correlates with outcome of hepatocellular carcinoma patients who underwent liver transplantation.
- This is the first study reported that hepatocellular carcinoma patients with HBV reactivation after liver transplantation are more likely to experience tumour recurrence.
- The nomogram based on Milan criteria, microvascular invasion and HBsAg-positive graft can effectively predict HBV reactivation after liver transplantation.

Hepatocellular carcinoma (HCC) is the sixth most common cancer in the world and related mortality ranks third^1,2^. In China, HCC is the leading cause of cancer-related death in males under 60 years old^3,4^. Liver transplantation is currently considered the optimal treatment option for patients with HCC^5–7^. However, HCC recurrence and metastasis seriously restrict the efficacy of liver transplantation. Tumour recurrence is frequently extrahepatic, with lung metastases accounting for 52.0–55.7%^8–10^. Moreover, the prognosis for HCC patients with extrahepatic metastasis remains poor and the median survival time is only 4.9–7.0 months^11,12^. Consequently, it is necessary to explore the potential factors associated with post-transplant lung metastasis.

Hepatitis B virus (HBV) infection is the major risk factor for the progression of liver cirrhosis and HCC, particularly in East Asia^13^. As a reflection of HBV infection status, hepatitis B seroepidemiology and HBV DNA have been often studied for their role in the prediction of postoperative prognosis^14–16^. However, few studies focus on the association between post-transplant HBV status and HCC recurrence and metastasis.

HBV reactivation is a frequent complication of immunosuppressive therapy given for malignancies, solid organ transplant, and inflammatory or autoimmune diseases^17^. The clinical consequences of liver injury caused by HBV reactivation vary greatly among individuals, ranging from asymptomatic liver function disturbances on blood tests to severe hepatitis, or even liver failure^18^. Besides, HBV reactivation may modulate the status of tumour biology in HCC patients^19^. Unfortunately, the risk of HBV reactivation after liver transplantation for HBV-related HCC is still underreported, and the relationship between post-transplant tumour recurrence and metastasis and HBV reactivation has not been investigated. This study aimed both to evaluate the incidence and the correlated factors for HBV reactivation and to determine the influence of HBV reactivation on tumour recurrence and metastasis after liver transplantation in HBV-related HCC.

## Materials and methods

### Patients

From January 2015 to December 2020, the electronic medical records of liver cancer patients who underwent liver transplantation at the two centres were retrospectively reviewed. The inclusion criteria were (1) preoperative diagnosis of HBV-related HCC and (2) pathologically confirmed HCC. The exclusion criteria were as follows: (1) non-HBV-related HCC; (2) simultaneous presence of other tumours; (3) re-transplantation; (4) presence of portal vein tumour thrombus; and (5) a survival time of less than 90 days. Patients who were lost to follow-up were also excluded. This retrospective study has been reported in line with the STROCSS criteria^20^, Supplemental Digital Content 1, http://links.lww.com/JS9/B889. Written informed consent to participate in this study was obtained from all patients. The study was reviewed and approved by the Ethics Committee of the two centres, and complied with the ethical guidelines of the Declaration of Helsinki.

### Management of patients

Anti-HBV therapy with nucleotide analogue was routinely performed for the patients on the diagnosis of HBV before transplantation. However, some patients are not aware of HBV infection until the diagnosis of HCC, and they may not have sufficient antivirus treatments before transplantation. Recipients were followed up after liver transplantation until 31 Dec 2022. During the follow‐up period, the recipients were managed according to a standard protocol. The prophylaxis of hepatitis B recurrence was routinely performed using a nucleoside analogue (entecavir/tenofovir) and hepatitis B immunoglobulin according to the clinical practice guideline on liver transplantation for hepatocellular carcinoma in China^4^. The follow‐up interval for all recipients was as long as 3 months in the stable phase after liver transplantation. HBV serological markers, such as hepatitis B surface antigen (HBsAg), hepatitis B e antigen (HBeAg) and HBV DNA were monitored regularly. The alpha-fetoprotein (AFP) level was determined every 1–2 months. The ultrasound or computed tomography or magnetic resonance imaging was performed every 3–6 months in HCC patients who underwent liver transplantation. Overall survival (OS) was defined as the number of months from the date of surgery to the date of the last follow-up visit or time of death. Recurrence-free survival (RFS) was defined as the number of months from the date of surgery to the date of first confirmable recurrence or death. Lung metastasis-free survival (LMFS) was defined as the number of months from the date of surgery to the date of first confirmable lung metastasis or death. HBV reactivation was defined as (a) HBsAg reappearance and (b) HBV DNA reappearance or increase greater than 10-fold^17,21,22^. HBV reactivation-free survival was defined as the number of months from the date of surgery to the date of HBV reactivation or death. HCC metastasis site was determined based on radiological evidence according to the standard guidelines for HCC.

### Statistical analysis

Statistical analysis was performed using GraphPad Prism (Version 9), IBM SPSS Statistics (Version 26) and R version 4.3.1 (R Foundation). For the baseline characteristics, continuous variables were presented as median interquartile range (IQR) values and compared by the Mann–Whitney U test. Categorical variables were compared using the chi-squared test or Fisher’s exact test.

Cumulative OS, RFS, LMFS and HBV reactivation-free rates were analyzed by the Kaplan–Meier method and compared using the log-rank test. To minimize potential confounders and selection bias, propensity score matching (PSM) was performed to construct comparable cohorts. Unbalanced variables were entered into the 1:2 matching model^23^. Cumulative incidence function curves were generated to estimate the influence of HBV reactivation on recurrence and death. Cox proportional hazard models were used to estimate hazard ratios (HRs) with 95%CIs for the relationship between variables and the event of interest. Univariate analyses were performed to identify the potential risk factors for OS, RFS, LMFS and HBV reactivation in each group. Variables (*P*<0.05) and interested variables were included in the forward stepwise multivariate analyses.

Results with *P* less than 0.05 were considered statistically significant. According to the independent risk factors of post-transplant HBV reactivation, nomogram, receiver operating characteristic curve (ROC) and calibration plots were established using the Sangerbox website^24^. The concordance index (C-index) and area under the time-dependent receiver operating characteristic curve (AUROC) were used to evaluate the discriminative ability. Calibration plots were used to evaluate the calibrating ability. The closer the C-index and AUROC values were to 1, the better the predicted results of the model.

## Results

### Patients’ characteristics

During the study period, there were 920 recipients with liver transplantation for liver cancer between January 2015 and December 2020. Four hundred and fifty-eight patients were excluded from the analysis, including 19 with re-transplantation, 2 who were missing follow‐up, 40 with intrahepatic cholangiocarcinoma, 19 with combined hepatocellular-cholangiocarcinoma, 252 with portal vein tumour thrombus, 85 with HBsAg negative and 41 with a survival time of less than 90 days. Ultimately, 462 patients who fulfilled the criteria were enroled in the final analysis (Supplemental Fig. 1, Supplemental Digital Content 2, http://links.lww.com/JS9/B890). Of the 462 patients, 421 (91.1%) were male, and the median age of the patients was 52 years (range 18–74 years; IQR 47–59 years). The median value of the laboratory MELD score was 25 (IQR 11–35). The median follow‐up duration for the whole population was 38.3 (IQR 26.4–50.2) months. The mean interval for post-transplant HCC recurrence in the study cohort was 8.4 (IQR 4.2–13.4) months. The 1-year recurrence rate, lung metastasis rate, liver metastasis rate and bone metastasis rate were 23.6%, 13.2%, 12.6% and 4.8%, respectively. The median follow‐up duration for the whole population was 38.3 (IQR 26.4–50.2) months. A total of 130 patients (28.1%) died during follow-up. The median time from transplantation to death was 21.8 months (IQR: 10.4–29.1 months). HCC recurrence was the most common cause of death [101 (21.9% of cohort, 77.7% of deaths)]. Severe infection and organ dysfunction were the cause of death in 13 patients (2.8% of cohort, 10.0% of deaths). Sixteen patients (3.4% of cohort, 12.3% of deaths) died for other reasons. The baseline clinical characteristics of the study population are summarized in Table 1.

**Table 1:** Baseline characteristics of 462 HCC patients undergoing liver transplantation.

Variable	Total (*n*=462)
Recipient age (years)	52 (47–59)
Recipient sex (*n*, % female)	41 (8.9)
Recipient BMI (kg/m^2^)	22.0 (20.5–23.9)
Pre-transplant AFP level (ng/ml)	22.0 (4.5−217.6)
Maximum tumour diameter (*n*, % >5cm)	131 (28.4)
Tumour number (*n*, % >3)	109 (23.6)
Tumour differentiation (*n*, % poor)	133 (28.8)
Microvascular invasion (*n*, %)	172 (37.2)
Liver cirrhosis (*n*, %)	431 (93.3)
Milan criteria (*n*, % beyond)	229 (49.6)
MELD at transplantation	25 (11–35)
Pre-transplant serum HBsAg (IU/ml)	513.9 (72.3–1328.3)
Pre-transplant serum HBeAg positive (*n*, %)	96 (20.8)
Pre-transplant HBV-DNA detectable (*n*, %)	211 (45.7)
Donor age (years)	47 (38–56)
Donor sex (*n*, % female)	73 (15.8)
Donor BMI (kg/m^2^)	22.9 (21.0–24.5)
HBsAg-positive graft (*n*, %)	81 (17.5)
Post-transplant recurrence (*n*, %)	158 (34.2)
Post-transplant lung metastasis (*n*, %)	94 (20.3)
Post-transplant liver metastasis (*n*, %)	87 (18.8)
Post-transplant bone metastasis (*n*, %)	43 (9.3)
Follow‐up (months)	38.3 (26.4–50.2)

AFP, alpha-fetoprotein; HBeAg, hepatitis B e antigen; HBsAg, hepatitis B surface antigen; HBV, hepatitis B virus; HCC, hepatocellular carcinoma.

### Dynamical change of post-transplant HBsAg and HBV DNA indicate different prognoses

**Figure 1:**
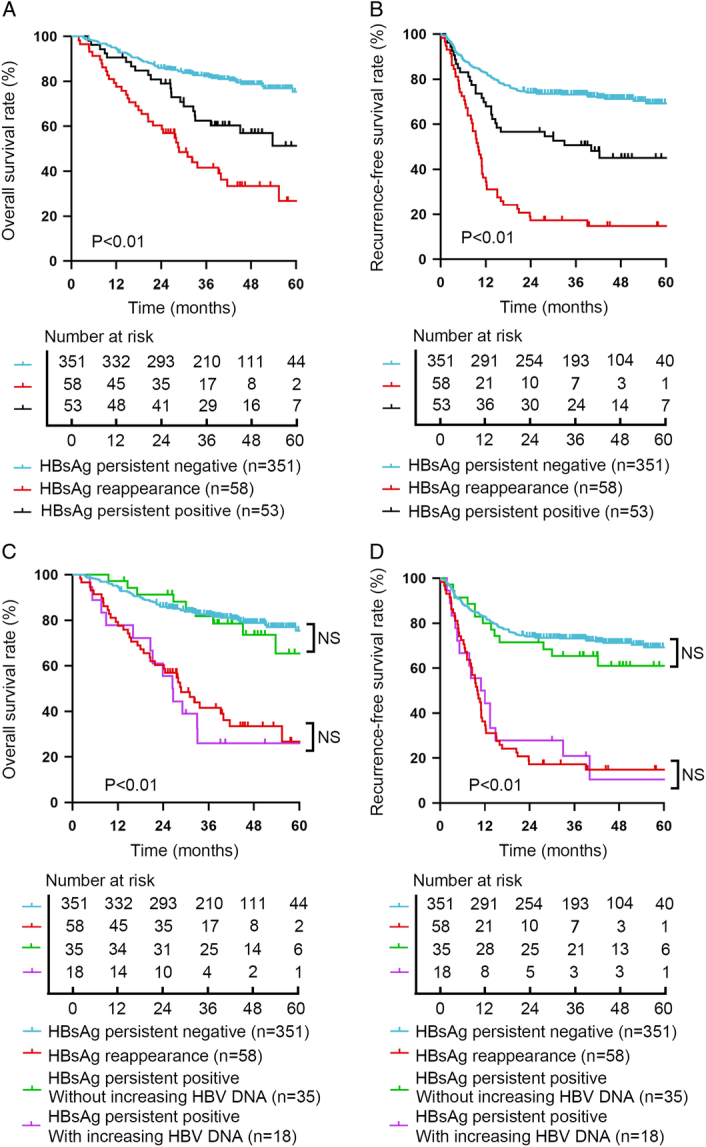
Cumulative incidence of survival. (A) Overall survival (OS) compared among different hepatitis B surface antigen (HBsAg) status. (B) Recurrence-free survival (RFS) compared among different HBsAg status. (C) OS compared among different HBsAg and hepatitis B virus (HBV) DNA status. (D) RFS compared among different HBsAg and HBV DNA status.

**Figure 2:**
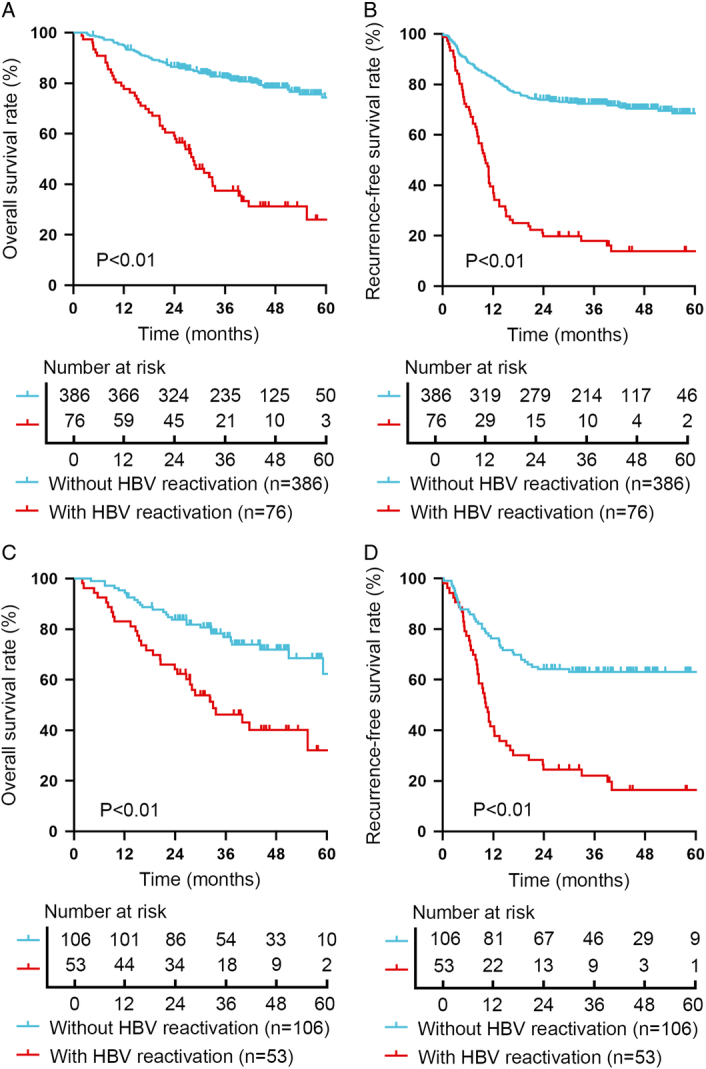
Clinical impacts of hepatitis B virus (HBV) reactivation. (A) Overall survival (OS) compared between different HBV reactivation status before propensity score matching (PSM). (B) Recurrence-free survival (RFS) compared between different HBV reactivation status before PSM. (C) OS compared between different HBV reactivation status after PSM. (D) RFS compared between different HBV reactivation status after PSM.

Based on the differential dynamical change of HBsAg after liver transplantation, patients were classified into HBsAg persistent negative group (*n*=351), HBsAg reappearance group (*n*=58) and HBsAg persistent positive group (*n*=53). Kaplan–Meier survival analysis showed that patients in the HBsAg reappearance group and the HBsAg persistent positive group had significantly worse OS and RFS than those in the HBsAg persistent negative group (Fig. 1A, B). The 1-year, 3-year and 5-year OS rates for patients in the HBsAg persistent negative group, the HBsAg reappearance group and the HBsAg persistent positive group were 94.9%, 82.3% and 75.4% vs. 77.6%, 41.6% and 26.7% vs. 90.6%, 62.5% and 51.3%, respectively (*P*<0.01, Fig. 2A). The 1-year, 3-year and 5-year RFS rates for patients in the HBsAg persistent negative group, the HBsAg reappearance group and the HBsAg persistent positive group were 82.9%, 73.1% and 69.3% vs. 36.2%, 17.2% and 14.8% vs. 67.9%, 50.6% and 45.1%, respectively (*P*<0.01, Fig. 1B).

Unexpectedly, we found that the prognosis of patients in the HBsAg persistent positive group was better than the HBsAg reappearance group. To further explore this surprising phenomenon, patients in HBsAg persistent positive group were further classified into two subgroups according to the differential changes of HBV DNA after liver transplantation. We defined increasing HBV DNA as HBV DNA reappearance and increase greater than 10-fold which represents exacerbation of chronic HBV infection. Kaplan–Meier survival analysis showed that patients in the HBsAg persistent positive with increasing HBV DNA subgroup had significantly worse overall survival and recurrence-free survival than those in the other subgroup without increasing HBV DNA (Fig. 1C, D).

Moreover, no significant difference was found in OS and RFS between patients in the HBsAg persistent negative group and the HBsAg persistent positive without increasing HBV DNA group. In the HBsAg persistent negative group, the 1‐year, 3‐year, and 5‐year OS rates were, respectively, 94.9%, 82.3% and 75.4% compared with 97.1%, 81.8%, and 65.5% for the HBsAg persistent positive without increasing HBV DNA group (Fig. 1C, D). The 1‐year, 3‐year, and 5‐year RFS rates for patients in the HBsAg persistent negative group were, respectively, 82.9%, 73.1% and 69.3% compared with 80.0%, 65.5%, and 61.1% for the HBsAg persistent positive without increasing HBV DNA group (Fig. 1C, D).

Coincidentally, no significant difference was also found in OS and RFS between patients in the HBsAg reappearance group and the HBsAg persistent positive with increasing HBV DNA group. In the HBsAg reappearance group, the 1‐year,3‐year, and 5‐year OS rates were, respectively, 77.6%, 41.6% and 26.7% compared with 77.8%, 25.9%, and 25.9% for the HBsAg persistent positive with increasing HBV DNA group (Fig. 1C-D). The 1‐year, 3‐year, and 5‐year RFS rates for patients in the HBsAg reappearance group were, respectively, 36.2%, 17.2% and 14.8% compared with 44.4%, 20.8%, and 10.4% for the HBsAg persistent positive with increasing HBV DNA group (Fig. 1C, D).

Therefore, we found that not only HBsAg reappearance but also HBsAg persistent positive with increasing HBV DNA is a hint of poor prognosis for HCC patients who underwent liver transplantation.

### Post-transplant HBV reactivation is associated with poor prognosis and a high risk of recurrence

Either HBsAg reappearance or increasing HBV DNA indicates that HBV replication is reactivated. Therefore, HBV reactivation was defined as (a) HBsAg reappearance and (b) HBV DNA reappearance or increase greater than 10-fold^17,21,22^. In this study cohort, the median HBV reactivation time was 7.9 (3.3–12.5) months and the 1-year HBV reactivation rate was 12.1%. Kaplan–Meier survival analysis showed that patients with HBV reactivation had significantly worse OS and RFS than those without HBV reactivation (*P*<0.01, Fig. 2A, B). The 1-year, 3-year and 5-year OS rates for patients without HBV reactivation and with HBV reactivation were 95.1%, 82.2% and 74.2% vs. 77.6%, 37.4% and 26.0%, respectively (Fig. 2A). The 1-year, 3-year and 5-year RFS rates for patients without HBV reactivation and with HBV reactivation were 82.6%, 72.4% and 68.5% vs. 38.2%, 17.9% and 17.9%, respectively (Fig. 2B).

However, patients with HBV reactivation had higher pre-transplant AFP levels, heavier tumour burden and more malignant biological properties than patients without HBV reactivation as shown in Table 2. To minimize the impacts of the above factors, PSM was used to construct more comparable cohorts. After 1:2 matching, the variables which can influence the patients’ prognosis were balanced as summarized in Supplemental Table 1, Supplemental Digital Content 3, http://links.lww.com/JS9/B891. Kaplan–Meier survival analysis still showed that patients with HBV reactivation had significantly worse OS and RFS than those without HBV reactivation (*P*<0.01, Fig. 2C, D). The 1-year, 3-year and 5-year OS rates for patients without HBV reactivation and with HBV reactivation were 95.3%, 76.8% and 62.3% vs. 83.0%, 46.2% and 32.0%, respectively (Fig. 2C). The 1-year, 3-year and 5-year RFS rates for patients without HBV reactivation and with HBV reactivation were 76.4%, 63.1% and 63.1% vs. 41.5%, 22.1% and 16.4%, respectively (Fig. 2D).

**Table 2:** Factors for HBV reactivation in HCC patients undergoing liver transplantation before PSM.

Variable	Without HBV reactivation (*n*=386)	With HBV reactivation (*n*=76)	*P*
Recipient age (years)	53 (47–59)	53 (47–59)	0.768
Recipient sex (*n*, % female)	38 (9.8)	3 (3.9)	0.098
Recipient BMI (kg/m^2^)	22.0 (20.6–23.9)	21.3 (20.5–23.4)	0.320
Pre-transplant AFP level (ng/ml)	19.6 (4.2–164.0)	77.7 (6.5–517.6)	0.005
Tumour max diameter (*n*, % >5cm)	99 (25.6)	32 (42.1)	0.004
Tumour number (*n*, % >3)	80 (20.7)	29 (38.2)	0.001
Tumour differentiation (*n*, % poor)	110 (28.5)	23 (30.3)	0.756
Microvascular invasion (*n*, %)	125 (32.4)	47 (61.8)	<0.001
Liver cirrhosis (*n*, %)	362 (93.8)	69 (90.8)	0.340
Milan criteria (*n*, % beyond)	177 (45.9)	52 (68.4)	<0.001
MELD at transplantation	23 (11–38)	32 (9–39)	0.301
Pre-transplant serum HBsAg (IU/mL)	417.9 (69.1–1148.6)	806.6 (77.2–1867.6)	0.058
Pre-transplant serum HBeAg positive (*n*, %)	77 (19.9)	19 (25.0)	0.321
Pre-transplant HBV-DNA detectable (*n*, %)	166 (43.0)	45 (59.2)	0.010
Donor age (years)	47 (38–55)	48 (37–57)	0.466
Donor sex (*n*, % female)	60 (15.5)	13 (17.1)	0.733
Donor BMI (kg/m^2^)	22.9 (21.2–24.5)	22.9 (20.8–24.5)	0.843
HBsAg-positive graft (*n*, %)	55 (14.2)	26 (34.2)	<0.001
Post-transplant recurrence (*n*, %)	98 (25.4)	60 (78.9)	<0.001
Post-transplant lung metastasis (*n*, %)	59 (15.3)	35 (46.1)	<0.001
Post-transplant liver metastasis (*n*, %)	49 (12.7)	38 (50.0)	<0.001
Post-transplant bone metastasis (*n*, %)	24 (6.2)	19 (25.0)	<0.001

AFP, alpha-fetoprotein; HBeAg, hepatitis B e antigen; HBsAg, hepatitis B surface antigen; HBV, hepatitis B virus; HCC, hepatocellular carcinoma; PSM, propensity score matching.

Considering that death was a competitive risk outcome, we generated cumulative incidence function curves to estimate the influence of HBV reactivation on recurrence and death. We found that patients with HBV reactivation had higher cumulative recurrence risk than patients without HBV reactivation after controlling death whether PSM or not (*P*<0.01, Supplemental Fig. 2 A, B, Supplemental Digital Content 4, http://links.lww.com/JS9/B892). In particular, the 5-year cumulative incidence of recurrence in patients with HBV reactivation was 80% compared with 26% in patients without HBV reactivation before PSM (Supplemental Fig. 2A, Supplemental Digital Content 4, http://links.lww.com/JS9/B892). The 5-year cumulative incidence of recurrence in patients with HBV reactivation was 76% compared with 33% in patients without HBV reactivation after PSM (Supplemental Fig. 2B, Supplemental Digital Content 4, http://links.lww.com/JS9/B892).

These data indicate that HCC patients who underwent liver transplantation with HBV reactivation had worse prognosis and higher risk of tumour recurrence than those without HBV reactivation.

### HBV reactivation was an independent risk factor of prognosis for HCC patients who underwent liver transplantation

To evaluate the potential risk factors of post-transplant OS, we performed univariate Cox regression analysis on the characteristics shown in Table 3. According to univariate and multivariate Cox regression analysis, we found that 5 variables including beyond Milan criteria, pre-transplant AFP greater than 400, poor tumour differentiation, microvascular invasion and HBV reactivation were independent risk factors for post-transplant OS (Table 3).

**Table 3:** Cox regression analysis of the variables of OS in 462 HCC patients after liver transplantation.

	Univariable predictors of OS			Multivariable predictors of OS		
Variable	*P*	HR	95% CI	*P*	HR	95% CI
Recipient age (years)	0.200	1.013	0.993–1.033			
Recipient sex (female)	0.928	0.972	0.524–1.803			
Recipient BMI (kg/m^2^)	0.299	0.969	0.913–1.028			
Beyond Milan criteria	<0.001	2.972	2.372–4.845	0.002	1.907	1.275–2.854
Pre-transplant AFP > 400 (ng/ml)	<0.001	2.700	1.870–3.897	0.001	1.900	1.302–2.773
Poor tumour differentiation	<0.001	1.949	1.372–2.770	0.008	1.632	1.137–2.342
Microvascular invasion	<0.001	2.993	2.105–4.257	0.004	1.756	1.203–2.563
MELD at transplantation	0.441	0.955	0.981–1.008			
Pre-transplant HBsAg (log IU/ml)	0.740	1.031	0.861–1.234			
Pre-transplant HBeAg positive	0.936	1.107	0.669–1.546			
Pre-transplant HBV-DNA detectable	0.322	1.191	0.843–1.682			
HBsAg-positive graft	0.071	1.456	0.968–2.191	0.929	0.981	0.645–1.492
HBV reactivation	<0.001	4.487	3.132–6.429	<0.001	3.467	2.380–5.052
Donor age (years)	0.108	1.010	0.998–1.023			
Donor sex (female)	0.984	0.995	0.618–1.603			
Donor BMI (kg/m^2^)	0.356	1.027	0.970–1.088			

AFP, alpha-fetoprotein; HBeAg, hepatitis B e antigen; HBsAg, hepatitis B surface antigen; HBV, hepatitis B virus; HCC, hepatocellular carcinoma; HR, hazard ratio; OS, overall survival.

Similarly, to evaluate the potential risk factors of post-transplant RFS, we performed univariate Cox regression analysis on the characteristics shown in Supplemental Table 2, Supplemental Digital Content 5, http://links.lww.com/JS9/B893. According to univariate Cox regression analysis, we found that 8 variables including recipient BMI, Milan criteria, pre-transplant AFP greater than 400, poor tumour differentiation, microvascular invasion, pre-transplant HBV DNA, HBsAg-positive graft and HBV reactivation were significantly correlated with RFS (all *P*<0.05, Supplemental Table 2, Supplemental Digital Content 5, http://links.lww.com/JS9/B893). The above variables were subsequently included in multivariate Cox regression analysis, and the results showed that beyond Milan criteria, pre-transplant AFP greater than 400, poor tumour differentiation, microvascular invasion and HBV reactivation were independent risk factors for post-transplant RFS (Supplemental Table 2, Supplemental Digital Content 5, http://links.lww.com/JS9/B893).

### HBV reactivation was related to post-transplant lung metastasis of patients with HCC

**Figure 3:**
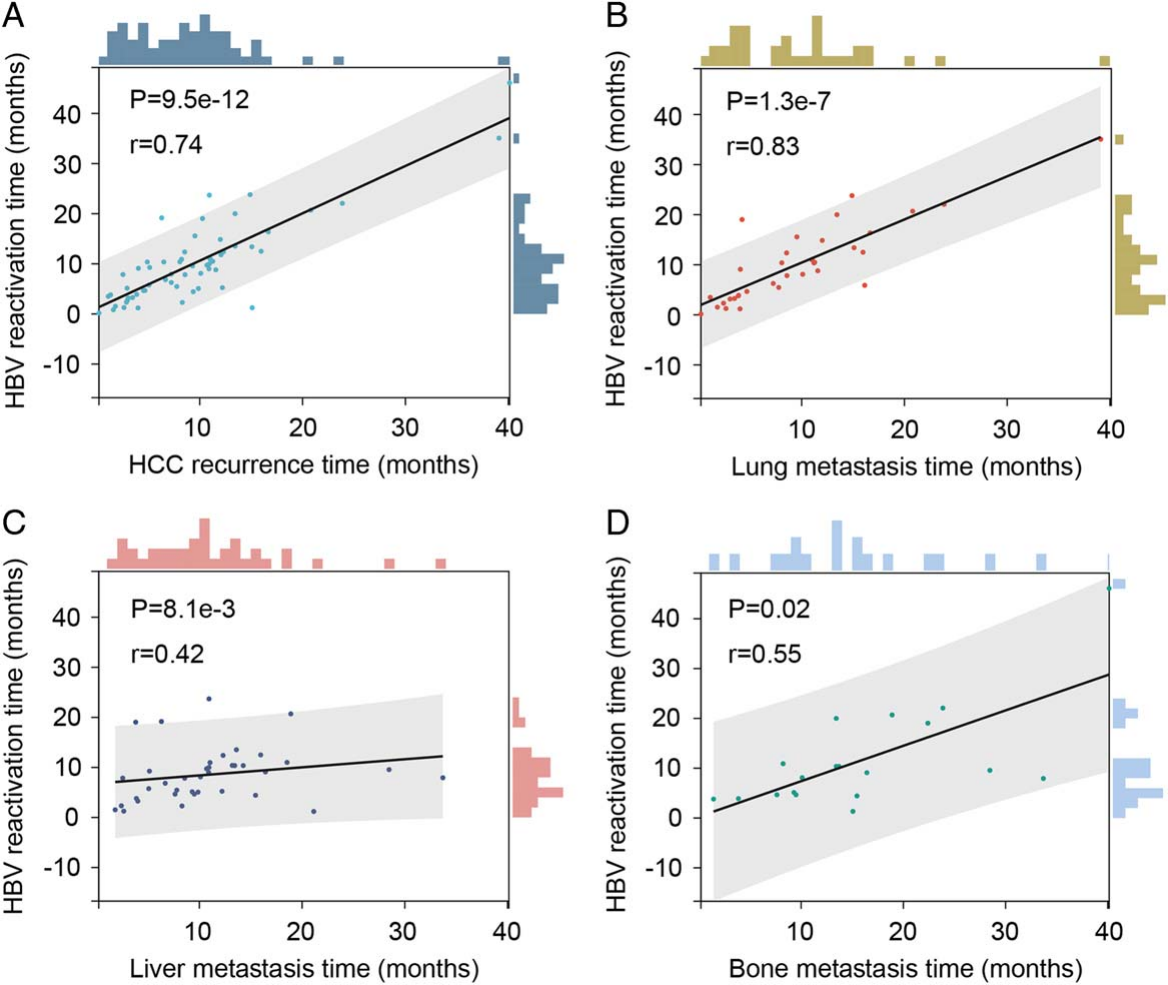
Correlation between hepatitis B virus (HBV) reactivation and hepatocellular carcinoma (HCC) recurrence times in patients with both HBV reactivation and HCC recurrence after liver transplantation. (A) The Spearman’s correlation between HBV reactivation and HCC recurrence time. (B) The Spearman’s correlation between HBV reactivation and lung metastasis time. (C) The Spearman’s correlation between HBV reactivation and liver metastasis time. (D) The Spearman’s correlation between HBV reactivation and bone metastasis time.

To determine whether the impact of HBV reactivation on HCC recurrence/metastasis would vary according to the site of metastasis, overall recurrence/metastasis was categorized into three groups: lung metastasis, liver metastasis and bone metastasis. Correlation analysis demonstrated a strong correlation between HBV reactivation and HCC recurrence (*P*=9.5e-12, r=0.74, Fig. 3). In addition, the correlation between HBV reactivation and lung metastasis (*P*=1.3e-7, r=0.83, Fig. 3) was strongest compared to liver metastasis (*P*=8.1e-3, r=0.42, Fig. 3) and bone metastasis (*P*=0.02, r=0.55, Fig. 3). In 35 patients with HBV reactivation and lung metastasis, the median HBV reactivation time was 9.1 (3.9–14.9) and the median lung metastasis time was 8.5 (IQR: 3.9–13.4) months. Twenty-two patients showed HBV reactivation first and 11 patients had lung metastasis first. Two other patients showed HBV reactivation and diagnosed lung metastasis on the same day.

To further evaluate the impact of HBV reactivation for post-transplant LMFS, we performed univariate Cox regression analysis on the characteristics shown in Supplemental Table 3, Supplemental Digital Content 6, http://links.lww.com/JS9/B894. According to univariate Cox regression analysis, we found that 8 variables including recipient BMI, beyond Milan criteria, pre-transplant AFP greater than 400, poor tumour differentiation, microvascular invasion, pre-transplant HBV DNA, HBsAg-positive graft and HBV reactivation were significantly correlated with LMFS (all *P*<0.05, Supplemental Table 3, Supplemental Digital Content 6, http://links.lww.com/JS9/B894). The above variables were subsequently included in multivariate Cox regression analysis, and the results showed that beyond Milan criteria, pre-transplant AFP greater than 400, poor tumour differentiation, microvascular invasion and HBV reactivation were independent risk factors for post-transplant LMFS (Supplemental Table 3, Supplemental Digital Content 6, http://links.lww.com/JS9/B894).

### A novel nomogram was established to predict post-transplant HBV reactivation of patients with HCC

To evaluate the potential risk factors of post-transplant HBV reactivation, we performed univariate Cox regression analysis on the characteristics shown in Table 4. According to univariate Cox regression analysis, we found that 5 variables including beyond Milan criteria, pre-transplant AFP greater than 400, microvascular invasion, pre-transplant HBV DNA detectable and HBsAg-positive graft were significantly correlated with HBV reactivation (all *P*<0.05, Table 4). The above variables were subsequently included in multivariate Cox regression analysis, and the results showed that beyond Milan criteria, microvascular invasion and HBsAg-positive graft were independent risk factors for post-transplant HBV reactivation (Table 4).

**Table 4:** Cox regression analysis of the variables of HBV reactivation-free survival in 462 HCC patients after liver transplantation.

	Univariable predictors of HBV reactivation-free survival			Multivariable predictor of HBV reactivation-free survival		
Variable	*P*	HR	95% CI	*P*	HR	95% CI
Recipient age (years)	0.777	1.004	0.979–1.029			
Recipient sex (female)	0.130	0.410	0.129–1.301			
Recipient BMI (kg/m^2^)	0.316	0.960	0.886350318			
Beyond Milan criteria	<0.001	2.619	1.628–4.212	0.015	1.852	1.125–3.048
Pre-transplant AFP > 400 (ng/ml)	0.014	1.879	1.136–3.109	0.476	1.211	0.715–2.053
Poor tumour differentiation	0.545	1.163	0.713–1.898			
Microvascular invasion	<0.001	3.187	2.005–5.066	<0.001	2.612	1.622–4.204
MELD at transplantation	0.109	1.015	0.997–1.033			
Pre-transplant HBsAg (log IU/ml)	0.155	1.027	0.931–1.563			
Pre-transplant HBeAg positive	0.340	1.288	0.766–2.164			
Pre-transplant HBV-DNA detectable	0.011	1.815	1.149–2.868	0.334	1.261	0.787–2.021
HBsAg-positive graft	<0.001	2.651	1.650–4.258	0.001	2.226	1.380–3.590
Donor age (years)	0.342	1.008	0.992–1.024			
Donor sex (female)	0.742	1.105	0.608–2.008			
Donor BMI (kg/m^2^)	0.961	0.998	0.928–1.074			
Recipient age (years)	0.777	1.004	0.979–1.029			

AFP, alpha-fetoprotein; HBeAg, hepatitis B e antigen; HBsAg, hepatitis B surface antigen; HBV, hepatitis B virus; HCC, hepatocellular carcinoma; HR, hazard ratio.

**Figure 4:**
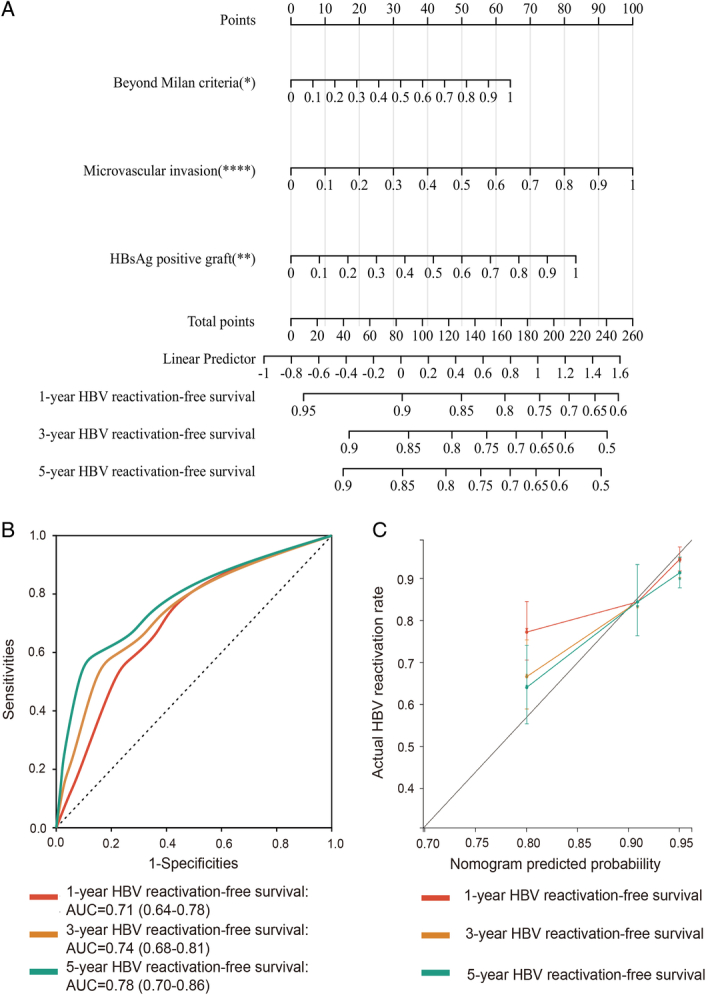
Nomogram for predicting post-transplant hepatitis B virus (HBV) reactivation and validation of its efficacy. (A) The nomogram to predict the 1-year, 3-year and 5-year HBV reactivation-free survival of hepatocellular carcinoma patients after liver transplantation; (B) Receiver operating characteristic curves of the nomogram; (C) Calibration curve of the nomogram. AUC, area under the curve; HBsAg, hepatitis B surface antigen.

According to the above 3 variables, we further established a novel nomogram for predicting the 1-year, 3-year and 5-year HBV reactivation-free survival rates of HCC patients who underwent liver transplantation (Fig. 4A). The C-index of the model was 0.73 (95% CI: 0.67−0.80). Time-dependent receiver operating characteristics (ROC) were used to evaluate the sensitivity and specificity of the model. We also illustrated the outcomes of ROC with the area under the curve (AUC). The AUROC of the 1-year, 3-year and 5-year HBV reactivation-free survival rates of HCC patients who underwent liver transplantation was 0.71 (95% CI: 0.64–0.78), 0.74 (95% CI: 0.68–0.81) and 0.78 (95% CI: 0.70–0.86), respectively (Fig. 4B). Calibration plots were used to confirm that the nomogram had good concordance with the prediction of 1-year, 3-year and 5-year HBV reactivation-free survival (Fig. 4C). To sum up, the nomogram for post-transplant HBV reactivation had considerable discriminative and calibration abilities.

### The nomogram can effectively predict the HBV reactivation and prognosis

According to the above nomogram, the risk score was calculated using the following formula: Risk score = 64.2 × Milan criteria (0, within Milan; 1, beyond Milan) + 100.0 × microvascular invasion (0, no; 1, yes) + 83.4 × HBsAg-positive graft (0, no; 1, yes). According to the number of risk factors, the entire cohort can be divided into low-risk group (0 risk factor, risk score=0, *n*=152), moderate-risk group (1 risk factor, 0<risk score<101, *n*=165) and high-risk group (2 or 3 risk factors, risk score greater than or equal to 101, *n*=145). The descriptive statistics for the three subgroups are listed in Supplemental Table 4, Supplemental Digital Content 7, http://links.lww.com/JS9/B895.

The 1-year, 3-year and 5-year cumulative HBV reactivation rates for patients in low-risk group, moderate-risk group and high-risk group were 4.0%, 6.6% and 6.6% vs. 10.9%, 12.7% and 12.7% vs 22.1%, 30.3% and 31.0%, respectively (Supplemental Fig. 3A, Supplemental Digital Content 8, http://links.lww.com/JS9/B896). In addition, the 1-year, 3-year and 5-year cumulative post-transplant lung metastasis rates for patients in low-risk group, moderate-risk group and high-risk group were 4.0%, 5.3% and 6.6% vs 11.5%, 16.4% and 17.0% vs. 27.6%, 38.0% and 38.6%, respectively (Supplemental Fig. 3B, Supplemental Digital Content 8, http://links.lww.com/JS9/B896).

Kaplan–Meier survival analysis showed a significant difference among these three subgroups (*P*<0.01). Especially, in comparison with the OS and RFS curve between the low-risk group and high-risk group, the OS and RFS of the low-risk group were statistically significantly higher than that of the high-risk group (85.3% 5-year OS rate in low-risk group versus 37.8% 5-year OS rate in high-risk group; 78.9% 5-year RFS rate in low-risk group versus 36.0% 5-year RFS rate in high-risk group) (Supplemental Fig. 3C, D, Supplemental Digital Content 8, http://links.lww.com/JS9/B896).

In summary, patients in low-risk group exhibited significantly lower HBV reactivation rates, post-transplant lung metastasis rates and better prognoses than patients in high-risk group.

## Discussion

In the previous study, post-transplant reverse HBsAg seroconversion (HBsAg negative becomes HBsAg positive) was significantly associated with the recipient’ worse survival^25^. Therefore, we assumed that the different changes in HBV status might be related to the prognosis of HBV-related HCC recipients who underwent liver transplantation. Not surprisingly, we found that HBsAg reappearance after liver transplantation was a hint of worse clinical outcomes. However, to our surprise, recipients with persistent HBsAg positive had a better prognosis than those with HBsAg reappearance. This interesting phenomenon reminds us of asymptomatic HBV carriers, whose lives are no different from healthy people due to HBV inactivity. HBV DNA, an indicator of the active status of HBV, was naturally included in our study. In the subsequent exploration, we found that HBsAg persistent positive recipients with HBV DNA reappearance or greater than 10-fold increase above baseline had worse prognosis than those without. Then we noticed that HBV reactivation perfectly illustrated the concept we wanted to propose, so we cited its relevant definition and further analyzed its role in liver transplantation for HCC.

HBV reactivation after various treatments of HCC has been well documented. It occurs in 7–19% of patients receiving surgical resection, transarterial chemoembolization, radiotherapy, local ablation therapy or systemic agents^26^. The clinical manifestation varies from an asymptomatic increase in HBV DNA level to fatal hepatic decompensation^26–28^. In this study, risk factors for HBV reactivation after liver transplantation on the univariate analysis included beyond Milan criteria, microvascular invasion, a detectable pre-transplant HBV DNA level and HBsAg-positive graft. A detectable pre-transplant HBV DNA level was not found to be an independent risk factor for reactivation of HBV replication on multivariate analysis, probably due to the small sample size.

In our study, the rate of HBV reactivation occurred in HCC patients after liver transplantation was 16.5%. On multivariate analysis, beyond Milan criteria, microvascular invasion and HBsAg-positive graft were independent risk factors for HBV reactivation. Several studies have reported that the presence of pre-transplant HCC is associated with post-transplant HBV recurrence^29,30^. We found that the Milan criterion based on HCC load was closely related to post-transplant HBV reactivation. Moreover, microvascular invasion, an important biological feature of HCC, was also associated with post-transplant HBV reactivation. Interestingly, both of the above factors were highly correlated with tumour recurrence after transplantation. Some scholars suggested that tumour recurrence increased the risk of HBV recurrence after transplantation^30^. Considering the detection of HBV DNA and covalently closed circular DNA (cccDNA) in HCC, they suggested that HBV replication in tumour cells may contribute to HBV recurrence^30,31^. We also found HCC recurrence can further increase the risk of HBV reactivation 5.14-fold, which may support this conjecture. In the previous study, we reported that HBsAg-positive grafts were related to increased risk of post-transplant HCC recurrence^32,33^. In this study, we further found that HBsAg-positive grafts elevated the risk of post-transplant HBV reactivation. Compared with HBsAg negative grafts, HBsAg-positive grafts reserved a high viral load of HBV, which might be an explanation for the phenomenon. Therefore, the rational use of HBsAg-positive grafts in clinic should be emphasized.

To precisely predict post-transplant HBV reactivation, we established a novel nomogram containing these independent risk factors. The predictive nomogram demonstrated good predictive efficacy. Moreover, based on the nomogram, we further stratified patients into low-risk, moderate-risk and high-risk groups. Patients in the high-risk group exhibited a higher risk of post-transplant lung metastasis and a worse prognosis than those in the low-risk group. Therefore, patients in high-risk group should be followed up and managed more closely to improve their prognosis as much as possible. In addition, prophylaxis with nucleos(t)ide analogues and hepatitis B immunoglobulin may bring benefits to this population^34^. Moreover, novel neutralizing antibodies, such as VIR-2218 and VIR-3434, might provide a new treatment for patients with chronic hepatitis B in the future^35,36^. The importance of optimal patient compliance with post-transplant antiviral prophylaxis should be also underlined^37^. The clinical practicality of our nomogram is expected to be validated in multicenter and large-sample cohorts.

The recurrence and metastasis of HCC after liver transplantation seriously restrict the efficacy of transplantation, in which the lung is the most common metastasis site^10,38^. Therefore, it is crucial to explore the factors related to lung metastasis after transplantation. In the previous study, we found that high tumour burden and pre-transplant HBsAg positive were the independent risk factors for post-transplant lung metastasis^39^. In this study, we further found that microvascular invasion and HBV reactivation were also independent risk factors for post-transplant lung metastasis. Microvascular invasion in HCC has been known as an independent predictor of tumour recurrence after surgical resection or liver transplantation^40,41^. Several studies have reported that patients with HBV reactivation after HCC resection were more likely to experience tumour recurrence^19,42,43^. Nevertheless, we believe we are the first group to report this issue in liver transplantation. HBV reactivation indicates HBV replication accelerating. Continuing HBV replication induces chronic liver injury and inflammation, which is associated with tumour recurrence. On one hand, the HBV X protein can promote the invasion and stemness of tumour cells, enhancing the colonization ability of tumour cells^44,45^. On the other hand, HBV-induced inflammation could inhibit the activation and function of NK cells and promote the polarization of macrophages from M1 to M2, creating a microenvironment suitable for tumour cell colonization^46–48^. However, the relevant researches mainly focus on the liver microenvironment other than the lung microenvironment at present. Further research on the mechanisms of HBV reactivation and post-transplant lung metastasis can provide reliable new methods and ideas for the prevention, diagnosis and treatment of HCC.

Further investigation of 35 patients concomitant with HBV reactivation and lung metastasis shows that there is a strong correlation between HBV reactivation and lung metastasis times. Twenty-two patients showed HBV reactivation first and 11 patients had lung metastasis first. Based on the consequence of the present study, we conjecture that HBV reactivation and lung metastasis may be reciprocal causation after liver transplantation. And their relationship still needs to be confirmed in a large cohort study.

There existed several limitations in this study. Firstly, lung metastasis after liver transplantation was diagnosed by serological testing and radiological examination. Despite the extremely low incidence of post-transplant primary lung cancer in HCC patients, the possibility cannot be completely ruled out. Secondly, the efficacy of the nomogram needed a larger multicenter cohort validation. Thirdly, as a retrospective observational study, it is impossible to follow random rules and avoid bias during analysis. Future prospective studies are needed to validate the conclusions. Finally, this study mainly focused on clinical phenomena, and further experiments are required to investigate the mechanism of the research.

## Conclusions

We demonstrated that HBV reactivation was highly associated with post-transplant HCC recurrence. Moreover, beyond Milan criteria, microvascular invasion and HBsAg-positive grafts are the independent risk factors of HBV reactivation. The nomogram deserves to be a helpful tool for predicting post-transplant HBV reactivation.

## Ethical approval

Ethical approvals for this study were provided by the Ethics Committee of the First Affiliated Hospital, Zhejiang University School of Medicine, Hangzhou, China (No.2020-510) and Ethics Committee of the Shulan (Hangzhou) Hospital, Hangzhou, China (No.KY2021014).

## Consent

Informed consent was waived as previously collected data that did not include personally identifiable information was used.

## Source of funding

This work was supported by the Key Research & Development Plan of Zhejiang Province (No.2024C03051), the Major Research Plan of the National Natural Science Foundation of China (No.92159202), the National Key Research and Development Program of China (No.2021YFA1100500), and the Health Science & Technology Plan of Zhejiang Province (No.2022RC060).

## Author contribution

X.X., S.Z. and L.Z. designed the research. H.L., D.L. and J.C. collected and organized data. H.L., D.L., J.C., J.Z., J.Z., Z.L., C.C. and W.S. analyzed the data. H.L., D.L., J.C., C.H., H.C. and Z.H. drafted the manuscript. Y.S., X.W., L.Z., S.Z. and X.X. contributed to the critical revision of the manuscript. All authors contributed to the manuscript and approved the submitted version.

## Conflicts of interest disclosure

The authors have no conflicts of interest to declare.

## Research registration unique identifying number (UIN)

1. Name of the registry: Clinical Trials.
2. Unique Identifying number or registration ID: NCT06114251.
3. Hyperlink to your specific registration (must be publicly accessible and will be checked): https://clinicaltrials.gov/ct2/show/NCT06114251.

## Guarantor

Xiao Xu.

## Data availability statement

The data that support the findings of this study are available on request from the corresponding author. The data are not publicly available due to privacy or ethical restrictions.

## Provenance and peer review

Not commissioned, externally peer-reviewed.

## Supplementary Material

**Figure:**
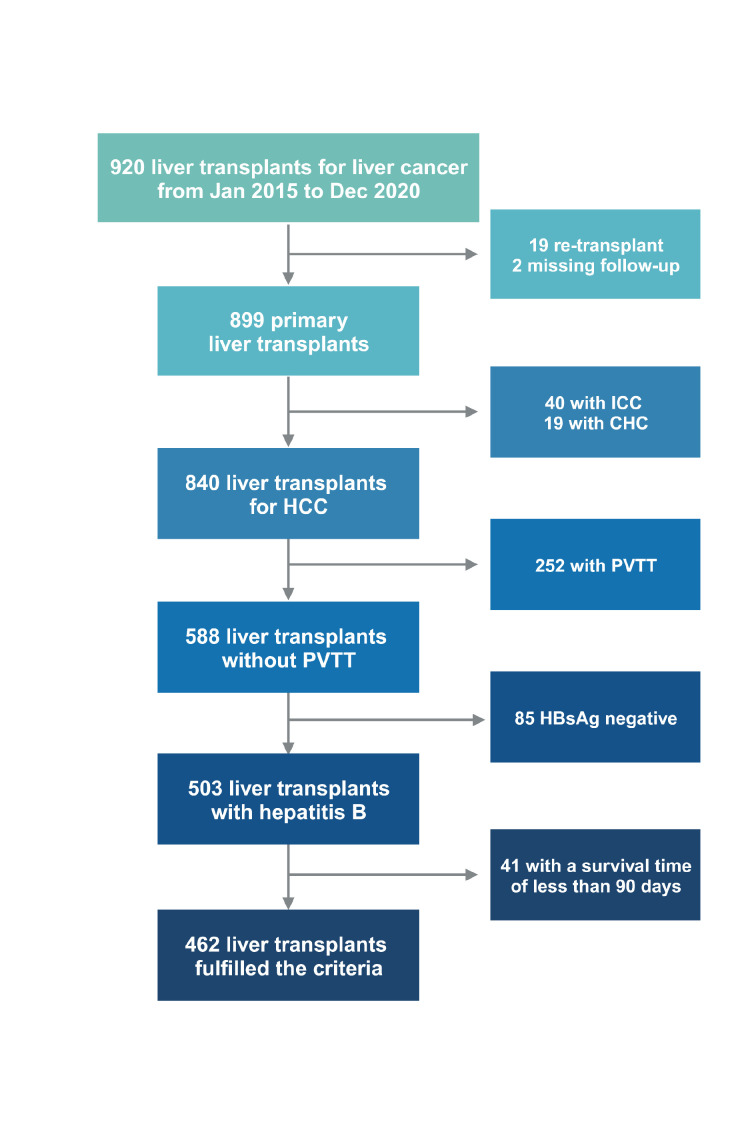


**Figure:**
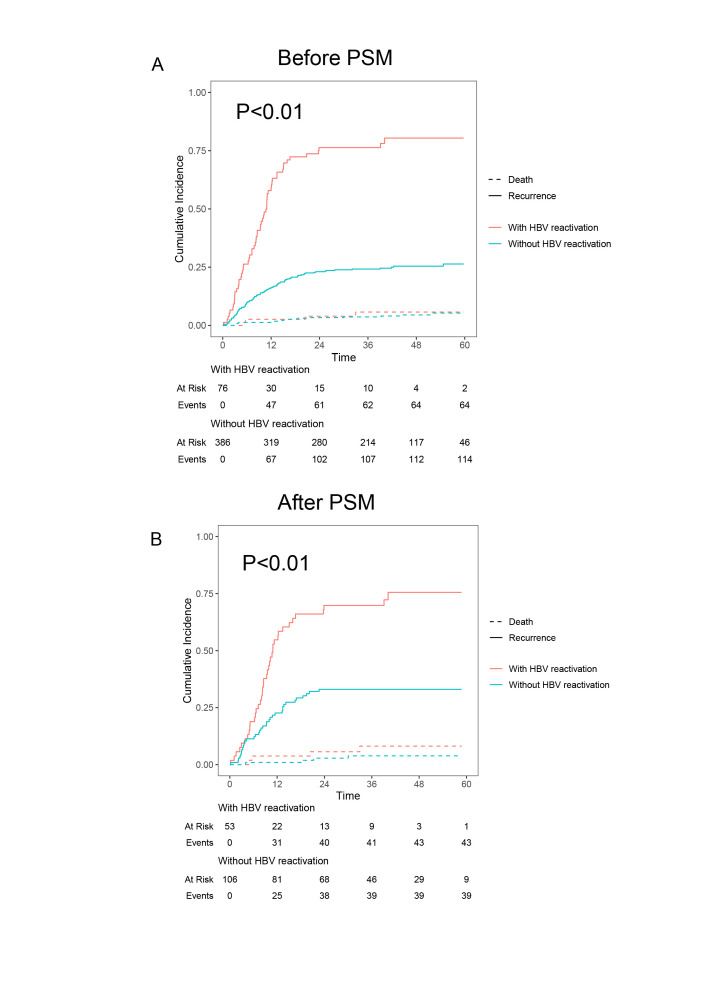


**Figure:**