# Supplementary material for: Post-transplant hepatitis B virus reactivation impacts the prognosis of patients with hepatitis B-related hepatocellular carcinoma: a dual-centre retrospective cohort study in China
Source: Int J Surg. 2024 Feb 9;110(4):2263–74. doi: 10.1097/JS9.0000000000001141 (PMC11019990; doi:10.1097/JS9.0000000000001141)
Supplement: Supplementary file 2 [file js9-110-2263-s003.docx]

| Supplemental Table 1.  Factors for HBV reactivation in HCC patients undergoing liver transplantation after PSM. | | | |
| --- | --- | --- | --- |
| Variable | Without HBV reactivation (n=106) | With HBV reactivation (n=53) | P-value |
| Recipient age (years) | 53 (47-59) | 53 (48-60) | 0.618 |
| Recipient sex (n, % female) | 9 (8.5%) | 1 (2.0%) | 0.106 |
| Recipient BMI (kg/m2) | 22.1 (20.7-24.1) | 21.3 (20.2-23.3) | 0.192 |
| Pre-transplant AFP level (ng/mL) | 21.1 (4.0-366.9) | 67.8 (6.2-276.1) | 0.342 |
| Tumor max diameter (n, % >5) | 35 (33.0%) | 18 (34.0%) | 0.905 |
| Tumor number (n, % >3) | 29 (27.4%) | 14 (26.4%) | 0.900 |
| Tumor differentiation (n, % poor) | 29 (27.4%) | 13 (24.5%) | 0.703 |
| Microvascular invasion (n, %) | 58 (54.7%) | 32 (60.4%) | 0.497 |
| Liver cirrhosis (n, %) | 100 (94.3%) | 47 (88.7%) | 0.203 |
| Milan criteria (n, % beyond) | 55 (51.9%) | 31 (58.5%) | 0.431 |
| MELD at transplantation | 20 (10-36) | 32 (10-39) | 0.129 |
| Pre-transplant serum HBsAg (IU/mL) | 350.5 (53.8-1178.2) | 766.0 (77.4-1858.7) | 0.225 |
| Pre-transplant serum HBeAg positive (n, %) | 25 (23.6%) | 12 (22.6%) | 0.894 |
| Pre-transplant HBV-DNA detectable (n, %) | 54 (50.9%) | 32 (60.4%) | 0.260 |
| Donor age (years) | 46 (37-55) | 49 (38-58) | 0.404 |
| Donor sex (n, % female) | 19 (17.9%) | 8 (15.1%) | 0.654 |
| Donor BMI (kg/m2) | 23.4 (20.8-24.2) | 22.9 (20.8-24.4) | 0.842 |
| HBsAg positive graft (n, %) | 25 (23.6%) | 15 (28.3%) | 0.518 |
| Post-transplant recurrence (n, %) | 36 (34.0%) | 39 (73.6%) | <0.001 |
| Post-transplant lung metastasis (n, %) | 20 (18.9%) | 24 (45.3%) | <0.001 |
| Post-transplant liver metastasis (n, %) | 20 (18.9%) | 22 (41.5%) | 0.002 |
| Post-transplant bone metastasis (n, %) | 10 (9.4%) | 14 (26.4%) | 0.005 |
