# Supplementary material for: Post-transplant hepatitis B virus reactivation impacts the prognosis of patients with hepatitis B-related hepatocellular carcinoma: a dual-centre retrospective cohort study in China
Source: Int J Surg. 2024 Feb 9;110(4):2263–74. doi: 10.1097/JS9.0000000000001141 (PMC11019990; doi:10.1097/JS9.0000000000001141)
Supplement: Supplementary file 3 [file js9-110-2263-s005.docx]

| Supplemental Table 2.  Cox regression analysis of the variables of RFS in 462 HCC patients after liver transplantation. | | | | | | | |
| --- | --- | --- | --- | --- | --- | --- | --- |
| Variable | Univariable predictors of RFS | | |  | Multivariable predictors of RFS | | |
|  | P value | HR | 95% CI |  | P value | HR | 95% CI |
| Recipient age (years) | 0.649 | 0.996 | 0.980-1.013 |  |  |  |  |
| Recipient sex (female) | 0.867 | 1.045 | 0.625-1.746 |  |  |  |  |
| Recipient BMI (kg/m2) | 0.044 | 0.947 | 0.899-0.999 |  | 0.799 | 0.993 | 0.942-1.047 |
| Beyond Milan criteria | <0.001 | 3.211 | 2.322-4.440 |  | <0.001 | 2.212 | 1.575-3.105 |
| Pre-transplant AFP > 400 (ng/ml) | <0.001 | 2.720 | 1.977-3.742 |  | <0.001 | 2.079 | 1.499-2.883 |
| Poor tumor differentiation | <0.001 | 1.845 | 1.363-2.497 |  | 0.007 | 1.531 | 1.121-2.093 |
| Microvascular invasion | <0.001 | 2.789 | 2.074-3.750 |  | 0.002 | 1.656 | 1.208-2.270 |
| MELD at transplantation | 0.184 | 1.008 | 0.996-1.020 |  |  |  |  |
| Pre-transplant HBsAg (log IU/mL) | 0.126 | 1.132 | 0.966-1.327 |  |  |  |  |
| Pre-transplant HBeAg positive | 0.290 | 1.204 | 0.854-1.696 |  |  |  |  |
| Pre-transplant HBV-DNA detectable | 0.008 | 1.485 | 1.107-1.991 |  | 0.483 | 0.896 | 0.659-1.218 |
| HBsAg positive graft | 0.048 | 1.427 | 1.004-2.029 |  | 0.678 | 0.925 | 0.639-1.338 |
| HBV reactivation | <0.001 | 4.841 | 3.538-6.623 |  | <0.001 | 3.768 | 2.710-5.239 |
| Donor age (years) | 0.878 | 0.999 | 0.989-1.010 |  |  |  |  |
| Donor sex (female) | 0.307 | 1.221 | 0.833-1.789 |  |  |  |  |
| Donor BMI (kg/m2) | 0.748 | 0.992 | 0.944-1.042 |  |  |  |  |
