# Supplementary material for: Post-transplant hepatitis B virus reactivation impacts the prognosis of patients with hepatitis B-related hepatocellular carcinoma: a dual-centre retrospective cohort study in China
Source: Int J Surg. 2024 Feb 9;110(4):2263–74. doi: 10.1097/JS9.0000000000001141 (PMC11019990; doi:10.1097/JS9.0000000000001141)
Supplement: Supplementary file 4 [file js9-110-2263-s006.docx]

| Supplemental Table 3.  Cox regression analysis of the variables of LMFS in 462 HCC patients after liver transplantation. | | | | | | | |
| --- | --- | --- | --- | --- | --- | --- | --- |
| Variable | Univariable predictors of LMFS | | |  | Multivariable predictors of LMFS | | |
|  | P value | HR | 95% CI |  | P value | HR | 95% CI |
| Recipient age (years) | 0.945 | 0.999 | 0.982-1.017 |  |  |  |  |
| Recipient sex (female) | 0.796 | 1.073 | 0.631-1.824 |  |  |  |  |
| Recipient BMI (kg/m2) | 0.049 | 0.946 | 0.895-1.000 |  | 0.567 | 0.983 | 0.929-1.041 |
| Beyond Milan criteria | <0.001 | 3.326 | 2.362-4.683 |  | <0.001 | 2.220 | 1.549-3.183 |
| Pre-transplant AFP > 400 (ng/ml) | <0.001 | 2.981 | 2.152-4.131 |  | <0.001 | 2.110 | 1.507-2.953 |
| Poor tumor differentiation | <0.001 | 1.879 | 1.371-2.575 |  | 0.026 | 1.446 | 1.046-2.000 |
| Microvascular invasion | <0.001 | 2.976 | 2.179-4.066 |  | 0.001 | 1.749 | 1.251-2.445 |
| MELD at transplantation | 0.292 | 1.007 | 0.994-1.019 |  |  |  |  |
| Pre-transplant HBsAg (log IU/mL) | 0.142 | 1.133 | 0.959-1.338 |  |  |  |  |
| Pre-transplant HBeAg positive | 0.260 | 1.227 | 0.859-1.754 |  |  |  |  |
| Pre-transplant HBV-DNA detectable | 0.007 | 1.53 | 1.123-2.081 |  | 0.902 | 0.980 | 0.713-1.348 |
| HBsAg positive graft | 0.050 | 1.442 | 1.000-2.081 |  | 0.968 | 0.992 | 0.677-1.453 |
| HBV reactivation | <0.001 | 4.225 | 3.053-5.846 |  | <0.001 | 3.009 | 2.144-4.223 |
| Donor age (years) | 0.686 | 1.002 | 0.991-1.014 |  |  |  |  |
| Donor sex (female) | 0.392 | 1.192 | 0.798-1.781 |  |  |  |  |
| Donor BMI (kg/m2) | 0.607 | 0.986 | 0.936-1.039 |  |  |  |  |
