# Supplementary material for: Post-transplant hepatitis B virus reactivation impacts the prognosis of patients with hepatitis B-related hepatocellular carcinoma: a dual-centre retrospective cohort study in China
Source: Int J Surg. 2024 Feb 9;110(4):2263–74. doi: 10.1097/JS9.0000000000001141 (PMC11019990; doi:10.1097/JS9.0000000000001141)
Supplement: Supplementary file 5 [file js9-110-2263-s007.docx]

| Supplemental Table 4.  Factors for the three risk groups stratified by the risk score in 462 HCC patients after liver transplantation. | | | | |
| --- | --- | --- | --- | --- |
| Variable | Low risk group (n=152) | Moderate risk group (n=165) | High risk group (n=145) | P value |
| Recipient age (years) | 54 (47-59) | 53 (47-58) | 53 (47-58) | 0.534 |
| Recipient sex (n, % female) | 20 (13.2%) | 11 (6.7%) | 10 (7.0%) | 0.076 |
| Recipient BMI (kg/m2) | 22.0 (20.8-23.9) | 22.4 (20.8-24.4) | 21.3 (20.3-23.2) | 0.015 |
| Pre-transplant AFP level (ng/mL) | 9.1 (3.4-83.6) | 19.2 (5.1-121.9) | 79.2 (88.7-1640.4) | <0.001 |
| Tumor max diameter (n, % >5) | 0 (0.0%) | 48 (29.1%) | 83 (57.2%) | <0.001 |
| Tumor number (n, % >3) | 0 (0.0%) | 36 (21.8%) | 73 (50.3%) | <0.001 |
| Tumor differentiation (n, % poor) | 28 (18.4%) | 49 (29.7%) | 56 (38.6%) | 0.001 |
| Microvascular invasion (n, %) | 0 (0.0%) | 48 (29.1%) | 124 (85.5%) | <0.001 |
| Milan criteria (n, % beyond) | 0 (0.0%) | 94 (57.0%) | 135 (93.1%) | <0.001 |
| MELD at transplantation | 21 (11-37) | 22 (10-38) | 31 (12-39) | 0.144 |
| Pre-transplant HBsAg (IU/mL) | 280.4  (53.6-1107.7) | 549.7  (71.5-1297.8) | 741.0  (88.7-1640.4) | 0.015 |
| Pre-transplant HBeAg positive (n, %) | 26 (17.1%) | 33 (20.0%) | 37 (25.5%) | 0.194 |
| Pre-transplant HBV-DNA detectable (n, %) | 42 (27.6%) | 78 (47.3%) | 91 (62.8%) | <0.001 |
| HBV reactivation | 10 (6.6%) | 21 (12.7%) | 45 (31.0%) | <0.001 |
| Donor age (years) | 47 (37-54) | 47 (37-55) | 47 (39-58) | 0.549 |
| Donor sex (n, % female) | 22 (14.4%) | 26 (15.8%) | 25 (17.2%) | 0.807 |
| Donor BMI (kg/m2) | 23.3 (21.5-25.2) | 22.9 (20.8-24.2) | 22.9 (20.8-24.2) | 0.138 |
| HBsAg positive graft (n, %) | 0 (0.0%) | 23 (13.9%) | 58 (40.0%) | <0.001 |
| Post-transplant recurrence (n, %) | 23 (15.1%) | 50 (30.3%) | 85 (58.6%) | <0.001 |
| Post-transplant lung metastasis (n, %) | 10 (6.6%) | 28 (17.0%) | 56 (38.6%) | <0.001 |
| Post-transplant liver metastasis (n, %) | 13 (8.6%) | 26 (15.8%) | 48 (33.1%) | <0.001 |
| Post-transplant bone metastasis (n, %) | 6 (4.0%) | 13 (7.9%) | 24 (16.6%) | 0.001 |
